# Impacto do Volume de Contraste Utilizado Após Procedimentos Coronários Percutâneos em Pacientes Predispostos à Nefropatia Induzida por Contraste

**DOI:** 10.36660/abc.20250270

**Published:** 2025-12-23

**Authors:** Rafaela Andrade Penalva Freitas, Luiz Fernando Leite Tanajura, Amanda Guerra M. R Sousa, Fausto Feres, José Ribamar Costa

**Affiliations:** 1 Instituto Dante Pazzanese de Cardiologia São Paulo SP Brasil Instituto Dante Pazzanese de Cardiologia, São Paulo, SP – Brasil; 2 Hospital São Domingos São Luís MA Brasil Hospital São Domingos, São Luís, MA – Brasil

**Keywords:** Nefropatias, Meios de Contraste, Intervenção Coronária Percutânea

## Abstract

**Fundamento:**

A nefropatia induzida por contraste (NIC) é complicação frequente após procedimentos angiográficos e pode estar relacionada ao volume de contraste administrado. Permanece incerto se o tipo de contraste também influencia sua ocorrência.

**Objetivo:**

Avaliar a interação entre volume e tipo de contraste (iso-osmolar ou de baixa osmolaridade) no desenvolvimento de NIC.

**Métodos:**

Subanálise post hoc de pacientes submetidos a procedimentos coronários diagnósticos e terapêuticos, randomizados 1:1 para contraste de baixa osmolaridade ou iso-osmolaridade. A amostra total (n = 2.268) foi estratificada por volume: Grupo I (< 150 ml; n = 1.985) e Grupo II (≥ 150 ml; n = 283), e comparada segundo o tipo de contraste. O desfecho primário foi NIC em 48 e 96 horas após o procedimento. NIC foi definida como elevação da creatinina sérica > 25% ou ≥ 0,5 mg/dl em relação ao nível basal após 48 horas. O efeito do tipo e do volume de contraste foi testado por regressão logística com termo de interação, ajustada para síndrome coronária aguda, disfunção ventricular, creatinina basal, sexo e idade (valor p < 0,05).

**Resultados:**

Foram incluídos 2.268 pacientes consecutivos; dois terços do sexo masculino; hipertensão arterial sistêmica em 85%, diabetes melito em 52% e doença renal crônica em 31%. No Grupo I, o volume médio de contraste foi 75,3 ± 28,0 ml; no Grupo II, 188,6 ± 46,9 ml. A incidência de NIC foi maior no grupo com maior volume (14,8% vs. 17,7%), sem significância estatística ( *odds ratio* ajustado = 1,25; intervalo de confiança de 95% 0,89-1,73; p = 0,191). O modelo com termo de interação não evidenciou correlação entre tipo de contraste e volume (p > 0,999).

**Conclusão:**

Não houve associação entre o tipo de contraste e a ocorrência de NIC, mesmo entre pacientes expostos a maiores volumes de contraste.

## Graphical abstract

**Figura Central:**
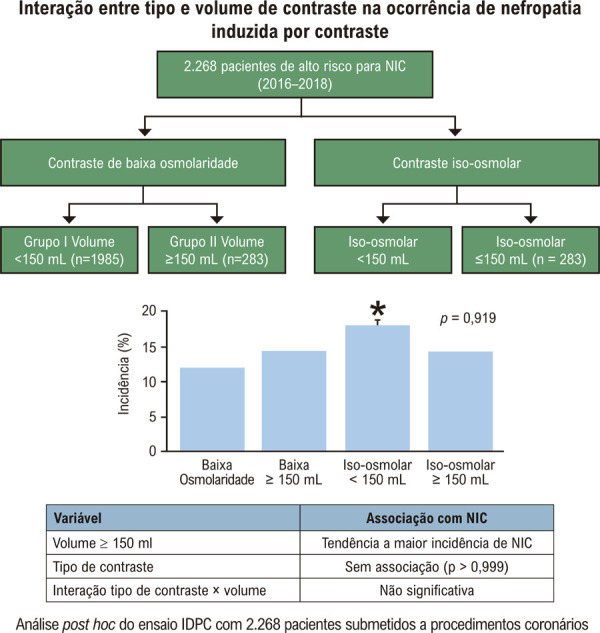
Impacto do Volume de Contraste Utilizado Após Procedimentos Coronários Percutâneos em Pacientes Predispostos à Nefropatia Induzida por Contraste

**Figura Central:** Impacto do Volume de Contraste Utilizado Após Procedimentos Coronários Percutâneos em Pacientes Predispostos à Nefropatia Induzida por Contraste

Impacto do Volume de Contraste Utilizado Após Procedimentos Coronários Percutâneos em Pacientes Predispostos à Nefropatia Induzida por Contraste.

## Introdução

A nefropatia induzida por contraste (NIC) caracteriza-se pelo surgimento de disfunção renal aguda após a administração de contraste endovenoso, na ausência de outras causas conhecidas de insuficiência renal aguda (IRA).^1 , 2^ É apontada como a terceira causa mais frequente de IRA em pacientes internados e associa-se a maior morbimortalidade e prolongamento do tempo de internação.^3^

A osmolaridade e o volume do contraste figuram entre os principais fatores associados ao desenvolvimento de NIC.^4^ Embora os mecanismos fisiopatológicos não estejam completamente elucidados, implicam-se efeitos diretos e indiretos do contraste, além de alterações hemodinâmicas inerentes aos procedimentos.^5^ A fisiopatologia da NIC resulta da sinergia entre toxicidade tubular e isquemia da medula renal.^5^ A injeção do contraste, além do efeito tóxico direto sobre o túbulo renal, altera mediadores da hemodinâmica renal — como prostaglandinas, óxido nítrico e adenosina — podendo precipitar isquemia.^5^ A medula renal externa, por apresentar pressão parcial de oxigênio relativamente baixa e elevada demanda metabólica, torna-se particularmente suscetível aos efeitos hemodinâmicos dos componentes do contraste.^5^

O ensaio *Ioxaglate Versus IoDixanol for the Prevention of Contrast-Induced Nephropathy* (IDPC; registro no ClinicalTrials.gov: NCT02991742) é, até o momento, o maior ensaio randomizado (n = 2.268) a comparar contrastes iso-osmolar (iodixanol) e de baixa osmolaridade (ioxaglato) quanto à redução de NIC após intervenção coronária percutânea (ICP) ou cateterismo diagnóstico. O principal achado foi a ausência de diferença significativa entre os dois tipos de contraste no desenvolvimento de NIC em população de alto risco submetida a procedimentos diagnósticos ou terapêuticos em laboratório de hemodinâmica de centro terciário. Do mesmo modo, não houve diferença significativa nos desfechos adversos combinados (óbito e hemodiálise) durante a internação e até 30 dias após o procedimento.

Destaca-se a baixa média de volume de contraste utilizada em nosso centro — uma rotina local —, comparável aos 5 anos anteriores ao estudo (89,0 ± 48,6 ml), sem variação significativa.^6^ Todos os pacientes, por serem de alto risco para NIC, seguiram protocolo de hidratação previamente descrito no estudo original.

Nesta subanálise, avaliamos se o volume de contraste utilizado em procedimentos percutâneos interage com o tipo de contraste (baixa vs. iso-osmolar) no desenvolvimento de NIC.

## Métodos

### Desenho do estudo e população avaliada

Este estudo é uma subanálise post hoc do ensaio IDPC, cujo desenho foi previamente detalhado e apresentado na Figura Central .^6^ Entre 2016 e 2018, todos os pacientes de alto risco para NIC submetidos à cinecoronariografia diagnóstica ou à ICP, em um único centro terciário da rede pública, foram incluídos e randomizados (1:1) para contraste iso-osmolar (iodixanol) ou de baixa osmolaridade (ioxaglato). No período, realizaram-se 16.244 procedimentos coronários percutâneos, dos quais 4.874 foram intervenções; a amostra deste estudo correspondeu a 14% do total. Consideraram-se de alto risco para NIC: idade ≥ 70 anos, insuficiência renal crônica (taxa de filtração glomerular [TGF] < 60 ml/min), diabetes melito, insuficiência cardíaca congestiva (ICC), choque ou uso de balão intra-aórtico, e procedimentos de urgência ou emergência.^6 , 7^

### Procedimentos do estudo

A coleta de dados ocorreu em três momentos. No dia do procedimento, registraram-se a administração do contraste iodado, o esquema de hidratação utilizado e as informações do procedimento angiográfico. Entre 48 e 96 horas após o procedimento, avaliaram-se sinais vitais, necessidade de diálise e níveis de creatinina sérica obtidos em consulta ambulatorial ou, quando o paciente permanecia internado, durante a internação; quando havia mais de uma medição nesse intervalo, considerou-se para análise o maior valor. Aos 30 dias, a equipe ambulatorial contatou os pacientes para verificar o estado de saúde, incluindo necessidade de diálise e condição vital. Além disso, imediatamente após a randomização, coletaram-se dados iniciais — características demográficas e clínicas — e o valor mais recente de creatinina sérica medido nas 48-96 horas anteriores.

A TFG foi calculada pela fórmula da Chronic Kidney Disease Epidemiology Collaboration.^8^ Pacientes com TFG ≤ 45 ml/min foram hospitalizados 1 dia antes do procedimento para hidratação intravenosa com solução salina 0,9% (0,5-1 ml/kg por hora, de acordo com a função ventricular esquerda), mantida por 12 horas após o procedimento; aqueles com disfunção renal receberam 5 ml/kg e os demais 1 ml/kg. Pacientes com TFG entre 45 e 60 ml/min também foram hidratados por 4 horas antes e 4 horas após o procedimento, sem necessidade de internação prévia. O protocolo de hidratação foi seguido de maneira uniforme em todos os pacientes, de forma semelhante entre os grupos.

### Desfecho primário

Nesta análise, avaliou-se a interação entre tipo de contraste (iso-osmolar vs. baixa osmolaridade) e volume utilizado durante os procedimentos. A população total foi estratificada em dois grupos: Grupo I (< 150 ml) e Grupo II (≥ 150 ml). O ponto de corte de 150 ml foi predefinido em protocolo para formar um grupo com maior exposição ao contraste e permitir melhor avaliação do efeito dessa variável. No estudo ACT, a mediana de contraste foi 100 ml e o terceiro quartil 130 ml.^7^

### Definições de desfechos

O desfecho primário foi a ocorrência de NIC, definida como elevação da creatinina sérica > 25% ou ≥ 0,5 mg/dl em relação ao valor basal, mensurada 48 horas após o procedimento.^6 , 9 - 11^ Os desfechos secundários incluíram o desfecho combinado de óbito por qualquer causa ou necessidade de diálise, conforme indicação da equipe de nefrologia, ocorridos durante a internação ou em até 30 dias após o procedimento, e óbito por todas as causas no mesmo período.

### Análise estatística

Por se tratar de análise post hoc, não houve cálculo formal do tamanho amostral, embora este estudo represente a maior avaliação contemporânea comparando tipos de contraste em cenário de maior volume utilizado. Realizou-se análise descritiva segundo a presença ou ausência de NIC. Variáveis contínuas foram resumidas por médias e desvios-padrão ou por medianas e intervalos interquartis, conforme a distribuição; variáveis categóricas foram apresentadas como frequências absolutas e relativas. As comparações entre grupos utilizaram o teste *t* de Student (contínuas) ou o teste do qui-quadrado (categóricas), excetuando-se creatinina, Cockcroft e TFG, comparadas pelo teste de Mann-Whitney. A normalidade foi avaliada por inspeção de histogramas e de gráficos Q-Q dos resíduos.

Para estimar o efeito do tipo e do volume de contraste sobre a ocorrência de NIC, empregou-se regressão logística com termo de interação, com ajuste para síndrome coronária aguda (SCA), disfunção ventricular, creatinina basal, sexo e idade. Adotou-se nível de significância de 5% (p < 0,05) para todas as análises.

## Resultados

A Tabela 1 apresenta a comparação das variáveis basais segundo o volume e o tipo de contraste utilizados. As Tabelas 2 e 3 mostram, respectivamente, a comparação das características basais conforme a ocorrência do desfecho (NIC vs. não NIC) e conforme a quantidade de contraste (< 150 ml vs. ≥ 150 ml).

**Tabela 1:** – Características iniciais dos pacientes por tipo de contraste e categoria de volume

Variável	Ioxaglato			Iodixanol		
	< 150 ml (n = 994)	≥ 150 ml (n = 139)	Valor p	< 150 ml (n = 991)	≥ 150 ml (n = 144)	Valor p
Idade, anos (média ± DP)	66,7 ± 10,8	67,1 ± 9,8	0,607	66,9 ± 10,5	65,6 ± 11,5	0,214
Sexo masculino, n (%)	612 (61,6)	91 (65,5)	0,402	629 (63,5)	107 (74,3)	0,012
IMC, kg/m^2^ (média ± DP)	28,7 ± 7,9	28,3 ± 5,9	0,468	28,3 ± 6,9	28,1 ± 5,6	0,637
Raça/cor, n (%)			0,948			0,680
Amarela	8 (0,8)	1 (0,7)		5 (0,5)	1 (0,7)	
Branca	738 (74,2)	103 (74,1)		728 (73,5)	112 (77,8)	
Parda	176 (17,7)	26 (18,7)		175 (17,7)	23 (16,0)	
Preta	56 (5,6)	8 (5,8)		71 (7,2)	6 (4,2)	
Mestiça	16 (1,6)	1 (0,7)		12 (1,2)	2 (1,4)	
Estatina, n (%)	771 (77,6)	107 (77,0)	0,914	753 (76,0)	108 (75,0)	0,835
BRA, n (%)	320 (32,2)	44 (31,7)	0,923	374 (37,7)	43 (29,9)	0,079
IECA, n (%)	409 (41,1)	68 (48,9)	0,099	378 (38,1)	58 (40,3)	0,647
Nitrato, n (%)	233 (23,4)	33 (23,7)	0,915	196 (19,8)	29 (20,1)	0,911
Fibrato, n (%)	16 (1,6)	2 (1,4)	0,999	16 (1,6)	3 (2,1)	0,724
BCC, n (%)	243 (24,4)	30 (21,6)	0,525	223 (22,5)	32 (22,2)	0,999
Diurético, n (%)	313 (31,5)	46 (33,1)	0,698	299 (30,2)	47 (32,6)	0,562
Digital, n (%)	11 (1,1)	1 (0,7)	0,999	9 (0,9)	0 (0,0)	0,613
Vasodilatador, n (%)	52 (5,2)	4 (2,9)	0,298	52 (5,2)	12 (8,3)	0,172
Insulina, n (%)	120 (12,1)	15 (10,8)	0,780	127 (12,8)	21 (14,6)	0,596
Hipoglicemiante oral, n (%)	418 (42,1)	54 (38,8)	0,521	381 (38,4)	66 (45,8)	0,100
HAS, n (%)	840 (84,5)	123 (88,5)	0,254	859 (86,7)	118 (81,9)	0,124
DM, n (%)	523 (52,6)	64 (46,0)	0,148	544 (54,9)	62 (43,1)	0,009
Dislipidemia, n (%)	665 (66,9)	91 (65,5)	0,773	633 (63,9)	96 (66,7)	0,577
Tabagismo, n (%)	400 (40,2)	59 (42,4)	0,645	359 (36,2)	50 (34,7)	0,781
IAM prévio, n (%)	326 (32,8)	43 (30,9)	0,700	340 (34,3)	57 (39,6)	0,225
ICP prévia, n (%)	137 (13,8)	13 (9,4)	0,181	138 (13,9)	21 (14,6)	0,798
CRM prévia, n (%)	131 (13,2)	20 (14,4)	0,690	134 (13,5)	29 (20,1)	0,041
História familiar positiva para DAC, n (%)	37 (3,7)	5 (3,6)	0,999	28 (2,8)	2 (1,4)	0,415
Obesidade, n (%)	244 (24,5)	27 (19,4)	0,203	207 (20,9)	28 (19,4)	0,742
Sedentarismo, n (%)	565 (56,8)	80 (57,6)	0,927	475 (47,9)	75 (52,1)	0,373
AVE prévio, n (%)	30 (3,0)	3 (2,2)	0,789	38 (3,8)	10 (6,9)	0,116
ICC, n (%)	51 (5,1)	7 (5,0)	0,999	79 (8,0)	9 (6,2)	0,616
Choque/BIA, n (%)	1 (0,1)	1 (0,7)	0,230	7 (0,7)	1 (0,7)	0,999
SCA, n (%)	383 (38,5)	63 (45,3)	0,138	371 (37,5)	62 (43,4)	0,197
TFG, ml/min/1,73 m^2^ (mediana [P25; P75])	73,0 [56,4; 91,6]	77,4 [57,0; 97,6]	0,132	72,3 [55,4; 92,1]	78,0 [62,8; 100,0]	0,008

AVE: acidente vascular encefálico; BCC: bloqueador de canal de cálcio; BIA: balão intra-aórtico; BRA: bloqueador do receptor de angiotensina; CRM: cirurgia de revascularização do miocárdio; DAC: doença arterial coronariana; DM: diabetes melito; DP: desvio padrão; HAS: hipertensão arterial sistêmica; IAM: infarto agudo do miocárdio; ICC: insuficiência cardíaca congestiva; ICP: intervenção coronária percutânea; IECA: inibidor da enzima conversora de angiotensina; IMC: índice de massa corporal; SCA: síndrome coronária aguda; TFG: taxa de filtração glomerular.

Na Tabela 2 , observa-se maior propensão à NIC entre pacientes com disfunção ventricular esquerda prévia, disfunção renal prévia e choque cardiogênico. Na Tabela 3 , destaca-se o uso mais frequente de volumes ≥ 150 ml em mulheres (70,0% vs. 62,5%; p = 0,015) e em pacientes com SCA (44,3% vs. 38,0%; p = 0,043), possivelmente em razão de procedimentos ad hoc (cateterismo seguido de ICP). Em contraste, volumes menores foram mais utilizados em pacientes com diabetes melito (44,5% vs. 53,8%; p = 0,040), refletindo cuidado adicional com essa população de muito alto risco.

**Tabela 2:** – Características iniciais dos pacientes segundo ocorrência de NIC

	NIC		
Variável	Ausente (n = 1.925)	Presente (n = 343)	Valor p
Idade, anos (média ± DP)	66,7 ± 10,7	67,2 ± 10,1	0,317
Sexo masculino, n (%)	1.202 (62,5)	236 (68,8)	0,028
IMC, kg/m^2^ (média ± DP)	28,5 ± 7,5	28,2 ± 5,5	0,322
Raça/cor, n (%)			0,231
Amarela	14 (0,7)	1 (0,3)	
Branca	1.410 (73,2)	271 (79,0)	
Parda	352 (18,3)	48 (14,0)	
Preta	122 (6,3)	19 (5,5)	
Mestiça	27 (1,4)	4 (1,2)	
Estatina, n (%)	1.492 (77,5)	247 (72,0)	0,032
BRA, n (%)	659 (34,2)	122 (35,6)	0,666
IECA, n (%)	781 (40,6)	132 (38,5)	0,474
Nitrato, n (%)	419 (21,8)	72 (21,0)	0,776
Fibrato, n (%)	34 (1,8)	3 (0,9)	0,352
BCC, n (%)	456 (23,7)	72 (21,0)	0,298
Diurético, n (%)	590 (30,6)	115 (33,5)	0,311
Digital, n (%)	14 (0,7)	7 (2,0)	0,029
Vasodilatador, n (%)	109 (5,7)	11 (3,2)	0,066
Insulina, n (%)	232 (12,1)	51 (14,9)	0,156
Hipoglicemiante oral, n (%)	763 (39,6)	156 (45,5)	0,049
HAS, n (%)	1.643 (85,4)	297 (86,6)	0,617
DM, n (%)	1.014 (52,7)	179 (52,2)	0,907
Dislipidemia, n (%)	1.265 (65,7)	220 (64,1)	0,579
Tabagismo, n (%)	749 (38,9)	119 (34,7)	0,148
IAM prévio, n (%)	646 (33,6)	120 (35,0)	0,620
ICP prévia, n (%)	272 (14,1)	37 (10,8)	0,105
CRM prévia, n (%)	263 (13,7)	51 (14,9)	0,553
História familiar positiva para DAC, n (%)	63 (3,3)	9 (2,6)	0,618
Obesidade, n (%)	428 (22,2)	78 (22,7)	0,833
Sedentarismo, n (%)	1.010 (52,5)	185 (53,9)	0,639
AVE prévio, n (%)	73 (3,8)	8 (2,3)	0,208
TFG 31-60 ml/min/1,73 m^2^, n (%)	561 (29,1)	125 (36,4)	0,007
Uso de fármaco vasoativo para IC, n (%)	114 (5,9)	32 (9,3)	0,023
Choque cardiogênico/BIA, n (%)	3 (0,2)	7 (2,0)	<0,001
SCA, n (%)	741 (38,5)	138 (40,2)	0,548
TFG, ml/min/1,73 m^2^ (mediana [P25; P75])	71,0 [54,1; 91,9]	68,1 [48,2; 92,9]	0,791

AVE: acidente vascular encefálico; BCC: bloqueador de canal de cálcio; BIA: balão intra-aórtico; BRA: bloqueador do receptor de angiotensina; CRM: cirurgia de revascularização do miocárdio; DAC: doença arterial coronariana; DM: diabetes melito; DP: desvio padrão; HAS: hipertensão arterial sistêmica; IAM: infarto agudo do miocárdio; ICC: insuficiência cardíaca congestiva; ICP: intervenção coronária percutânea; IECA: inibidor da enzima conversora de angiotensina; IMC: índice de massa corporal; SCA: síndrome coronária aguda; TFG: taxa de filtração glomerular.

**Tabela 3:** – Características iniciais dos pacientes segundo volume.

Variável	< 150 ml (n = 1.985)	≥ 150 ml (n = 283)	Valor p
Idade, anos (média ± DP)	66,8±10,6	66,4±10,7	0,543
Sexo masculino, n (%)	1.241 (62,5)	198 (70,0)	0,015
IMC, kg/m^2^ (média ± DP)	28,5±7,4	28,2±5,7	0,389
Raça/cor, n (%)			0,871
Amarela	13 (0,7)	2 (0,7)	
Branca	1.466 (73,9)	215 (76,0)	
Parda	351 (17,7)	49 (17,3)	
Preta	127 (6,4)	14 (4,9)	
Mestiça	28 (1,4)	3 (1,1)	
Estatina, n (%)	1.524 (76,8)	215 (76,0)	0,764
BRA, n (%)	694 (35,0)	87 (30,7)	0,181
IECA, n (%)	787 (39,6)	126 (44,5)	0,120
Nitrato, n (%)	429 (21,6)	62 (21,9)	0,939
Fibrato, n (%)	32 (1,6)	5 (1,8)	0,802
BCC, n (%)	466 (23,5)	62 (21,9)	0,599
Diurético, n (%)	612 (30,8)	93 (32,9)	0,493
Digital, n (%)	20 (1,0)	1 (0,4)	0,504
Vasodilatador, n (%)	104 (5,2)	16 (5,7)	0,776
Insulina, n (%)	247 (12,4)	36 (12,7)	0,923
Hipoglicemiante oral, n (%)	799 (40,3)	120 (42,4)	0,518
HAS, n (%)	1.699 (85,6)	241 (85,2)	0,857
DM, n (%)	1.067 (53,8)	126 (44,5)	0,004
Dislipidemia, n (%)	1.298 (65,4)	187 (66,1)	0,841
Tabagismo, n (%)	759 (38,2)	109 (38,5)	0,948
IAM prévio, n (%)	666 (33,6)	100 (35,3)	0,546
ICP prévia, n (%)	275 (13,9)	34 (12,0)	0,459
CRM prévia, n (%)	265 (13,4)	49 (17,3)	0,080
História familiar positiva para DAC, n (%)	65 (3,3)	7 (2,5)	0,588
Obesidade, n (%)	451 (22,7)	55 (19,4)	0,223
Sedentarismo, n (%)	1.040 (52,4)	155 (54,8)	0,484
AVE prévio, n (%)	68 (3,4)	13 (4,6)	0,306
Choque/BIA, n (%)	8 (0,4)	2 (0,7)	0,360
SCA, n (%)	754 (38,0)	125 (44,3)	0,043
TFG, ml/min/1,73 m^2^ (mediana [P25; P75])	72,6 [55,7; 91,8]	78,0 [59,7; 100,0]	0,003

AVE: acidente vascular encefálico; BCC: bloqueador de canal de cálcio; BIA: balão intra-aórtico; BRA: bloqueador do receptor de angiotensina; CRM: cirurgia de revascularização do miocárdio; DAC: doença arterial coronariana; DM: diabetes melito; DP: desvio padrão; HAS: hipertensão arterial sistêmica; IAM: infarto agudo do miocárdio; ICP: intervenção coronária percutânea; IECA: inibidor da enzima conversora de angiotensina; IMC: índice de massa corporal; SCA: síndrome coronária aguda; TFG: taxa de filtração glomerular.

Ajustou-se um modelo de regressão logística com termo de interação entre tipo de contraste e volume, a fim de testar se o efeito do contraste depende do volume administrado. O modelo foi também ajustado por idade, sexo, creatinina basal, ICC e SCA ( Tabela 4 ). O termo de interação não foi significativo (p > 0,05), indicando ausência de modificação de efeito pelo volume.

**Tabela 4:** – Modelos com termo de interação para nefropatia induzida por contraste

Variáveis	Modelo com interação, OR (IC 95%)	Valor p	Modelo com interação ajustado^**1**^, OR (IC 95%)	Valor p
Contraste				
Ioxaglato	—	—	—	—
Iodixanol	0,96 (0,75-1,24)	0,773	0,94 (0,73-1,20)	0,618
Volume de contraste				
< 150 ml	—	—	—	—
≥ 150 ml	1,07 (0,64-1,71)	0,796	1,07 (0,64-1,71)	0,796
Interação (Iodixanol × ≥ 150 ml)	1,33 (0,69-2,60)	0,397	1,35 (0,70-2,65)	0,377

^1^Ajustado para sexo, idade, creatinina basal, insuficiência cardíaca congestiva e síndrome coronária aguda. IC: intervalo de confiança; OR: odds ratio.

Ao remover o termo de interação, não se identificou efeito independente do tipo de contraste ou do volume sobre a ocorrência de NIC. A Tabela 5 mostra a tendência para maior risco com volumes ≥ 150 ml no modelo não ajustado ( *odds ratio* [OR] = 1,24; IC 95% 0,88-1,71; p = 0,202) e no modelo ajustado (OR = 1,25; IC 95% 0,89-1,73; p = 0,191). Análises adicionais tratando o volume como variável contínua também não demonstraram associação significativa com NIC.

**Tabela 5:** – Modelos aditivos para nefropatia induzida por contraste

Variáveis	Modelo aditivo, OR (IC 95%)	Valor p	Modelo aditivo ajustado^**1**^, OR (IC 95%)	Valor p
Contraste				
Ioxaglato	—	—	—	—
Iodixanol	1,00 (0,80-1,26)	0,974	0,98 (0,78-1,23)	0,857
Volume de contraste				
< 150 ml	—	—	—	—
≥ 150 ml	1,24 (0,88-1,71)	0,202	1,25 (0,89-1,73)	0,191

^1^Ajustado para sexo, idade, creatinina basal, insuficiência cardíaca congestiva e síndrome coronária agud

**Figura 1:**
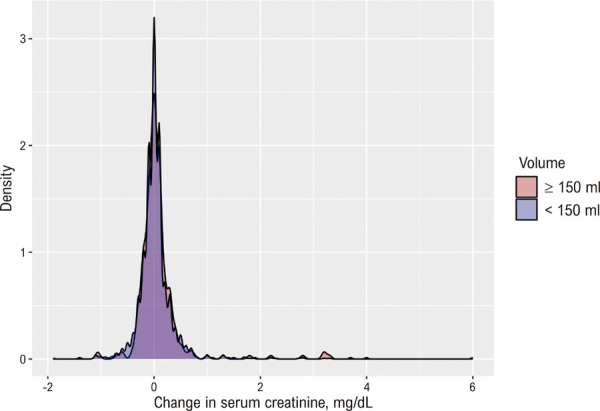
– Densidade da variação da creatinina por categoria de volume de contraste.

As Figuras 1 e 2 ilustram a ausência de relação entre o volume de contraste e a variação da creatinina. A Figura 3 apresenta o efeito de interação entre volume e tipo de contraste na NIC: a interação não foi significativa no modelo sem ajuste (OR = 1,33; IC 95% 0,69-2,60; p = 0,397) nem no modelo ajustado por sexo, idade, creatinina basal, ICC e SCA (OR = 1,35; IC 95% 0,70-2,65; p = 0,377).

**Figura 2:**
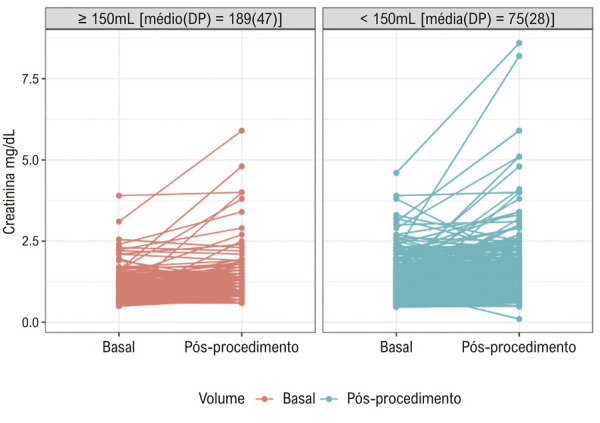
– Dispersão da variação da creatinina por categoria de volume de contraste. DP: desvio padrão.

**Figura 3:**
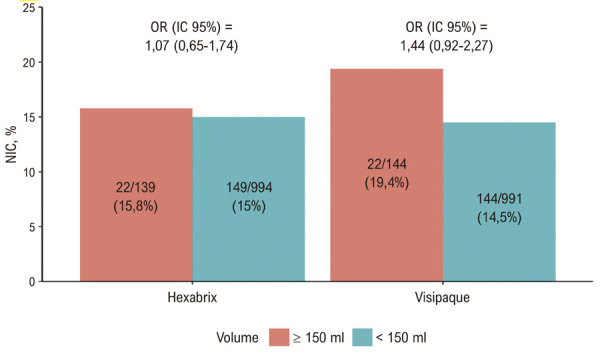
– Incidência de NIC por tipo de contraste e categoria de volume. NIC: nefropatia induzida por contraste; OR: odds ratio.

## Discussão

A NIC permanece uma preocupação relevante em procedimentos diagnósticos e terapêuticos que utilizam contraste iodado, particularmente na ICP.^9^ No presente estudo, o tipo de contraste (iso-osmolar vs. baixa osmolaridade) não se associou à ocorrência de NIC; o elemento crítico parece ser a minimização do volume administrado e a hidratação prévia em populações de alto risco.

A literatura sugere papéis relevantes para tipo e volume do contraste no risco de NIC.^9 , 10^ Contudo, não observamos diferenças significativas entre contrastes iso-osmolar e de baixa osmolaridade, independentemente do volume, lembrando que apenas uma minoria recebeu volumes realmente elevados nesta análise. Trabalhos mais antigos apontavam potencial vantagem dos agentes iso-osmolares por menor estresse hemodinâmico e renal,^11 , 12^ mas evidências recentes indicam que, em pacientes cuidadosamente selecionados e submetidos a protocolos adequados de hidratação, a escolha do agente pode não alterar de forma significativa a incidência de NIC. Ademais, a osmolaridade não é o único determinante da resposta renal ao contraste.

A ausência de diferença entre os grupos pode refletir a efetividade das estratégias preventivas implementadas. A hidratação intravenosa, amplamente recomendada em diretrizes, associada ao emprego de volumes reduzidos, provavelmente mitigou qualquer impacto do tipo de contraste sobre a função renal.

A heterogeneidade clínica também merece consideração. Fatores como diabetes melito, idade avançada e doença renal prévia — embora ajustados nas análises — podem modular individualmente o risco de NIC e contribuir para a ausência de efeito global.

Em termos práticos, a escolha do agente de contraste deve ser individualizada, ponderando condições clínicas, perfil de risco e disponibilidade local de recursos. Estudos prospectivos, com amostras maiores e delineamentos robustos, são necessários para confirmar estes achados e explorar diferenças potenciais em subgrupos específicos.

### Limitações do estudo

Além da osmolaridade e do volume, outras propriedades dos meios de contraste podem influenciar a ocorrência de NIC; portanto, os resultados não devem ser extrapolados para contrastes distintos dos dois avaliados. Ademais, trata-se de ensaio de centro único, o que limita a generalização dos achados para serviços que utilizem volumes mais elevados de contraste ou atendam populações diferentes.

## Conclusão

O tipo de contraste não se associou de forma significativa à ocorrência de NIC, mesmo entre pacientes expostos a maiores volumes de contraste.
