# Supplementary material for: Impacto do Volume de Contraste Utilizado Após Procedimentos Coronários Percutâneos em Pacientes Predispostos à Nefropatia Induzida por Contraste
Source: Arq Bras Cardiol. 2025 Dec 23;122(12):e20250270. [Article in Portuguese] doi: 10.36660/abc.20250270 (PMC12978361; doi:10.36660/abc.20250270)
Supplement: Translation

# Translation: Impact of Contrast Volume Used After Percutaneous Coronary Procedures in Patients at Risk for Contrast-Induced Nephropathy

**DOI:** 10.36660/abc.20250270i

**Authors:** Rafaela Andrade Penalva Freitas, Luiz Fernando Leite Tanajura, Amanda Guerra M. R Sousa, Fausto Feres, José Ribamar Costa

**Affiliations:** 1 Brazil Instituto Dante Pazzanese de Cardiologia, São Paulo, SP – Brazil; 2 Brazil Hospital São Domingos, São Luís, MA – Brazil

**Keywords:** Kidney Diseases, Contrast Media, Percutaneous Coronary Intervention

## Abstract

**Background:**

Contrast-induced nephropathy (CIN) is a common complication following angiographic procedures, potentially related to the volume of contrast agent administered. However, it is unclear whether the type of contrast also influences its occurrence.

**Objective:**

To evaluate the interaction between contrast volume and contrast type (iso-osmolar vs low-osmolar) in the development of CIN.

**Methods:**

A post hoc subanalysis was performed on patients undergoing diagnostic or therapeutic coronary procedures who were randomized 1:1 to receive low-osmolar or iso-osmolar contrast media. The overall sample (n = 2,268) was stratified by contrast volume: Group I (< 150 mL; n = 1,985) and Group II (≥ 150 mL; n = 283), and groups were compared by contrast type. The primary endpoint was CIN at 48- and 96-hours post-procedure. CIN was defined as a ≥ 25% or ≥ 0.5 mg/dL increase in serum creatinine from baseline at 48 hours. The effects of contrast type and volume were tested with logistic regression including an interaction term, adjusted for acute coronary syndrome, ventricular dysfunction, baseline creatinine, sex, and age (p < 0.05).

**Results:**

We included 2,268 consecutive patients; two-thirds were male; hypertension was present in 85%, diabetes in 52%, and chronic kidney disease in 31%. Mean contrast volume was 75.3 ± 28.0 mL in Group I and 188.6 ± 46.9 mL in Group II. The incidence of CIN was higher with greater volume (14.8% vs 17.7%) but not statistically significant (adjusted odds ratio = 1.25; 95% CI = 0.89-1.73; p = 0.191). The model with the interaction term showed no evidence of an interaction between the type of contrast and volume (p > 0.999).

**Conclusion:**

The type of contrast was not associated with CIN, even among patients exposed to higher contrast volumes.

## Introduction

Contrast-induced nephropathy (CIN) is defined as acute kidney dysfunction that occurs after the intravenous administration of iodinated contrast agents, in the absence of other known causes of acute kidney injury (AKI).^1 , 2^CIN is the third most common cause of AKI among hospitalized patients, and it is associated with higher rates of morbidity and mortality as well as a longer hospital stay.^3^

Among the main determinants of CIN are contrast osmolarity and administered volume.^4^Although the pathophysiology is not fully elucidated, both direct and indirect effects of contrast media — as well as procedure-related hemodynamic alterations — are implicated.^5^CIN arises from the synergy between tubular toxicity and ischemia of the renal medulla.^5^In addition to direct tubular toxicity, contrast injection perturbs renal hemodynamic mediators — such as prostaglandins, nitric oxide, and adenosine — thereby precipitating ischemia.^5^The outer renal medulla, which operates at relatively low oxygen tension and high metabolic demand, is particularly vulnerable to these hemodynamic effects.^5^

The *Ioxaglate Versus IoDixanol for the Prevention of Contrast-Induced Nephropathy* (IDPC; ClinicalTrials.gov NCT02991742) trial is, to date, the largest randomized study (n = 2,268) comparing iso-osmolar (iodixanol) versus low-osmolar (ioxaglate) contrast media for preventing CIN after percutaneous coronary intervention (PCI) or diagnostic catheterization. Its primary finding was the absence of a significant difference between contrast types in the incidence of CIN in a high-risk population undergoing diagnostic or therapeutic procedures in a tertiary catheterization laboratory. Likewise, there were no significant differences in the composite adverse outcomes (death and hemodialysis) during hospitalization or within 30 days post-procedure.

A notable feature of our center is the consistently low average contrast volume used—a local practice—comparable to the 5 years preceding the trial (89.0 ± 48.6 mL), with no significant variation.^6^All patients, because of their high risk for CIN, received hydration according to the protocol described in the original study.

In this post hoc subanalysis, we evaluated whether the volume of contrast used during percutaneous procedures interacts with contrast type (low- vs iso-osmolar) in the development of CIN.

## Methods

### Study design and population

This post hoc subanalysis derives from the IDPC trial, whose design has been detailed previously and is summarized in Central Illustration .^6^From 2016 to 2018, all high-risk patients for CIN undergoing diagnostic coronary angiography or PCI at a single tertiary public center were enrolled and randomized (1:1) to iso-osmolar (iodixanol) or low-osmolar (ioxaglate) contrast. During the period, 16,244 percutaneous coronary procedures were performed, including 4,874 interventions; the present sample accounted for 14% of all procedures. High-risk criteria for CIN were age ≥ 70 years, chronic kidney disease (estimated glomerular filtration rate [eGFR] < 60 mL/min), diabetes, congestive heart failure, shock or use of intra-aortic balloon, and urgency/emergency procedures.^6 , 7^

### Study procedures

Data were collected at three time points. On the procedure day, we recorded iodinated contrast administration, the hydration regimen, and angiographic procedural details. Between 48 and 96 hours after the procedure, vital signs, dialysis requirement, and serum creatinine levels were assessed at an outpatient visit or, when applicable, during hospitalization; when multiple measurements were available within this window, the highest value was used for analysis. At 30 days, clinic staff contacted patients to ascertain health status, including dialysis requirement and vital status. Immediately after randomization, baseline demographic and clinical characteristics and the most recent serum creatinine value measured within the prior 48-96 hours were obtained.

eGFR was calculated using the equation developed by the Chronic Kidney Disease Epidemiology Collaboration (CKD-EPI).^8^Patients with eGFR ≤ 45 mL/min were admitted 1 day before the procedure for intravenous hydration with 0.9% saline at 0.5-1 mL/kg per hour (according to left ventricular function), continued for 12 hours post-procedure; those with renal dysfunction received 5 mL/kg and the remainder 1 mL/kg. Patients with eGFR 45-60 mL/min also received hydration for 4 hours before and 4 hours after the procedure without pre-procedure admission. The hydration protocol was applied uniformly to all patients and similarly across randomized groups.

### Primary endpoint

This analysis evaluated the interaction between contrast type (iso-osmolar vs low-osmolar) and the contrast volume used during procedures. The overall cohort was stratified into two prespecified groups: Group I (< 150 mL) and Group II (≥ 150 mL). The 150-mL cutoff was defined a priori to delineate a group with higher exposure and to enable clearer assessment of volume effects. For reference, in the ACT study the median contrast volume was 100 mL and the third quartile 130 mL.^7^

### Outcome definitions

The primary endpoint was the occurrence of CIN, defined as an increase in serum creatinine of > 25% or ≥ 0.5 mg/dL from baseline, measured 48 hours after the procedure.^6 , 9 - 11^Secondary endpoints included a composite of all-cause death or the need for dialysis (as determined by the nephrology team) occurring during hospitalization or within 30 days post-procedure as well as all-cause mortality within the same period.

### Statistical analysis

As this was a post hoc analysis, no formal sample size calculation was performed; however, this study represents the largest contemporary assessment comparing contrast types in a high-volume exposure scenario. A descriptive analysis was conducted according to the presence or absence of CIN. Continuous variables were summarized as means and standard deviations or as medians and interquartile ranges, depending on their distribution; categorical variables were presented as absolute and relative frequencies. Between-group comparisons used the Student’s t-test for continuous variables and the chi-square test for categorical variables, except for creatinine, Cockcroft, and eGFR, which were compared using the Mann-Whitney test. Normality was evaluated through histogram inspection and Q-Q plots of residuals.

To estimate the effects of contrast type and volume on CIN occurrence, logistic regression including an interaction term was used, adjusted for acute coronary syndrome (ACS), ventricular dysfunction, baseline creatinine, sex, and age. A two-sided p-value < 0.05 was considered statistically significant for all analyses.

## Results

Table 1 summarizes baseline characteristics by contrast volume and type. Tables 2 and 3 present, respectively, baseline comparisons by outcome (CIN vs no CIN) and by contrast volume (< 150 mL vs ≥ 150 mL).

**Table 1:** – Baseline characteristics by contrast type and volume category

Variable	Ioxaglate < 150 mL (n = 994)	Ioxaglate ≥ 150 mL (n = 139)	p value	Iodixanol < 150 mL (n = 991)	Iodixanol ≥ 150 mL (n = 144)	p value
Age, years (mean ± SD)	66.7 ± 10.8	67.1 ± 9.8	0.607	66.9 ± 10.5	65.6 ± 11.5	0.214
Male sex, n (%)	612 (61.6)	91 (65.5)	0.402	629 (63.5)	107 (74.3)	0.012
BMI, kg/m^2^(mean ± SD)	28.7 ± 7.9	28.3 ± 5.9	0.468	28.3 ± 6.9	28.1 ± 5.6	0.637
Race/skin color, n (%)			0.948			0.680
Yellow	8 (0.8)	1 (0.7)		5 (0.5)	1 (0.7)	
White	738 (74.2)	103 (74.1)		728 (73.5)	112 (77.8)	
Brown	176 (17.7)	26 (18.7)		175 (17.7)	23 (16.0)	
Black	56 (5.6)	8 (5.8)		71 (7.2)	6 (4.2)	
Mixed	16 (1.6)	1 (0.7)		12 (1.2)	2 (1.4)	
Statin, n (%)	771 (77.6)	107 (77.0)	0.914	753 (76.0)	108 (75.0)	0.835
ARB, n (%)	320 (32.2)	44 (31.7)	0.923	374 (37.7)	43 (29.9)	0.079
ACEi, n (%)	409 (41.1)	68 (48.9)	0.099	378 (38.1)	58 (40.3)	0.647
Nitrate, n (%)	233 (23.4)	33 (23.7)	0.915	196 (19.8)	29 (20.1)	0.911
Fibrate, n (%)	16 (1.6)	2 (1.4)	0.999	16 (1.6)	3 (2.1)	0.724
CCB, n (%)	243 (24.4)	30 (21.6)	0.525	223 (22.5)	32 (22.2)	0.999
Diuretic, n (%)	313 (31.5)	46 (33.1)	0.698	299 (30.2)	47 (32.6)	0.562
Digitalis, n (%)	11 (1.1)	1 (0.7)	0.999	9 (0.9)	0 (0.0)	0.613
Vasodilator, n (%)	52 (5.2)	4 (2.9)	0.298	52 (5.2)	12 (8.3)	0.172
Insulin, n (%)	120 (12.1)	15 (10.8)	0.780	127 (12.8)	21 (14.6)	0.596
Oral hypoglycemic, n (%)	418 (42.1)	54 (38.8)	0.521	381 (38.4)	66 (45.8)	0.100
Hypertension, n (%)	840 (84.5)	123 (88.5)	0.254	859 (86.7)	118 (81.9)	0.124
Diabetes, n (%)	523 (52.6)	64 (46.0)	0.148	544 (54.9)	62 (43.1)	0.009
Dyslipidemia, n (%)	665 (66.9)	91 (65.5)	0.773	633 (63.9)	96 (66.7)	0.577
Current/former smoking, n (%)	400 (40.2)	59 (42.4)	0.645	359 (36.2)	50 (34.7)	0.781
Prior MI, n (%)	326 (32.8)	43 (30.9)	0.700	340 (34.3)	57 (39.6)	0.225
Prior PCI, n (%)	137 (13.8)	13 (9.4)	0.181	138 (13.9)	21 (14.6)	0.798
Prior CABG, n (%)	131 (13.2)	20 (14.4)	0.690	134 (13.5)	29 (20.1)	0.041
Positive family history of CAD, n (%)	37 (3.7)	5 (3.6)	0.999	28 (2.8)	2 (1.4)	0.415
Obesity, n (%)	244 (24.5)	27 (19.4)	0.203	207 (20.9)	28 (19.4)	0.742
Physical inactivity, n (%)	565 (56.8)	80 (57.6)	0.927	475 (47.9)	75 (52.1)	0.373
Prior stroke, n (%)	30 (3.0)	3 (2.2)	0.789	38 (3.8)	10 (6.9)	0.116
CHF, n (%)	51 (5.1)	7 (5.0)	0.999	79 (8.0)	9 (6.2)	0.616
Shock/IABP use, n (%)	1 (0.1)	1 (0.7)	0.230	7 (0.7)	1 (0.7)	0.999
ACS, n (%)	383 (38.5)	63 (45.3)	0.138	371 (37.5)	62 (43.4)	0.197
eGFR, mL/min/1.73 m^2^(median [P25; P75])	73.0 [56.4; 91.6]	77.4 [57.0; 97.6]	0.132	72.3 [55.4; 92.1]	78.0 [62.8; 100.0]	0.008

ACE: angiotensin-converting enzyme inhibitor; ACS: acute coronary syndrome; ARB: angiotensin receptor blocker; BMI: body mass index; CABG: coronary artery bypass grafting; CAD: coronary artery disease; CCB: calcium-channel blocker; CHF: congestive heart failure; eGFR: estimated glomerular filtration rate; IABP: intra-aortic balloon pump; ICP/PCI: percutaneous coronary intervention; MI: myocardial infarction; SD: standard deviation.

In Table 2 , CIN occurred more often among patients with prior left ventricular dysfunction, pre-existing renal dysfunction, and cardiogenic shock. In Table 3 , use of ≥ 150 mL was more frequent in women (70.0% vs 62.5%; p = 0.015) and in patients with ACS (44.3% vs 38.0%; p = 0.043), likely reflecting ad hoc procedures (diagnostic catheterization followed by PCI). Conversely, lower volumes were more common in patients with diabetes mellitus (44.5% vs 53.8%; p = 0.040), suggesting additional caution in this very-high-risk group.

**Table 2:** – Baseline characteristics by CIN occurrence

Variable	No CIN (n = 1,925)	CIN (n = 343)	p value
Age, years (mean ± SD)	66.7 ± 10.7	67.2 ± 10.1	0.317
Male sex, n (%)	1,202 (62.5)	236 (68.8)	0.028
BMI, kg/m^2^(mean ± SD)	28.5 ± 7.5	28.2 ± 5.5	0.322
Race/skin color, n (%)			0.231
Yellow	14 (0.7)	1 (0.3)	
White	1,410 (73.2)	271 (79.0)	
Brown	352 (18.3)	48 (14.0)	
Black	122 (6.3)	19 (5.5)	
Mixed	27 (1.4)	4 (1.2)	
Statin, n (%)	1,492 (77.5)	247 (72.0)	0.032
ARB, n (%)	659 (34.2)	122 (35.6)	0.666
ACEi, n (%)	781 (40.6)	132 (38.5)	0.474
Nitrate, n (%)	419 (21.8)	72 (21.0)	0.776
Fibrate, n (%)	34 (1.8)	3 (0.9)	0.352
CCB, n (%)	456 (23.7)	72 (21.0)	0.298
Diuretic, n (%)	590 (30.6)	115 (33.5)	0.311
Digitalis, n (%)	14 (0.7)	7 (2.0)	0.029
Vasodilator, n (%)	109 (5.7)	11 (3.2)	0.066
Insulin, n (%)	232 (12.1)	51 (14.9)	0.156
Oral hypoglycemic, n (%)	763 (39.6)	156 (45.5)	0.049
Hypertension, n (%)	1,643 (85.4)	297 (86.6)	0.617
Diabetes, n (%)	1,014 (52.7)	179 (52.2)	0.907
Dyslipidemia, n (%)	1,265 (65.7)	220 (64.1)	0.579
Current/former smoking, n (%)	749 (38.9)	119 (34.7)	0.148
Prior MI, n (%)	646 (33.6)	120 (35.0)	0.620
Prior PCI, n (%)	272 (14.1)	37 (10.8)	0.105
Prior CABG, n (%)	263 (13.7)	51 (14.9)	0.553
Positive family history of CAD, n (%)	63 (3.3)	9 (2.6)	0.618
Obesity, n (%)	428 (22.2)	78 (22.7)	0.833
Physical inactivity, n (%)	1,010 (52.5)	185 (53.9)	0.639
Prior stroke, n (%)	73 (3.8)	8 (2.3)	0.208
eGFR 31-60 mL/min/1.73 m^2^, n (%)	561 (29.1)	125 (36.4)	0.007
Use of vasoactive drugs for CHF, n (%)	114 (5.9)	32 (9.3)	0.023
Cardiogenic shock / IABP, n (%)	3 (0.2)	7 (2.0)	<0.001
ACS, n (%)	741 (38.5)	138 (40.2)	0.548
eGFR, mL/min/1.73 m^2^(median [P25; P75])	71.0 [54.1; 91.9]	68.1 [48.2; 92.9]	0.791

ACEi: angiotensin-converting enzyme inhibitor; ACS: acute coronary syndrome; ARB: angiotensin receptor blocker; CABG: coronary artery bypass grafting; CAD: coronary artery disease; CCB: calcium-channel blocker; CHF: congestive heart failure; CIN: contrast-induced nephropathy; eGFR: estimated glomerular filtration rate; IABP: intra-aortic balloon pump; MI: myocardial infarction; PCI: percutaneous coronary intervention; SD: standard deviation.

**Table 3:** – Baseline characteristics by contrast volume

Variable	< 150 mL (n = 1,985)	≥ 150 mL (n = 283)	p value
Age, years (mean ± SD)	66.8 ± 10.6	66.4 ± 10.7	0.543
Male sex, n (%)	1,241 (62.5)	198 (70.0)	0.015
BMI, kg/m^2^(mean ± SD)	28.5 ± 7.4	28.2 ± 5.7	0.389
Race/skin color, n (%)			0.871
Yellow	13 (0.7)	2 (0.7)	
White	1,466 (73.9)	215 (76.0)	
Brown	351 (17.7)	49 (17.3)	
Black	127 (6.4)	14 (4.9)	
Mixed	28 (1.4)	3 (1.1)	
Statin, n (%)	1,524 (76.8)	215 (76.0)	0.764
ARB, n (%)	694 (35.0)	87 (30.7)	0.181
ACE inhibitor, n (%)	787 (39.6)	126 (44.5)	0.120
Nitrate, n (%)	429 (21.6)	62 (21.9)	0.939
Fibrate, n (%)	32 (1.6)	5 (1.8)	0.802
CCB, n (%)	466 (23.5)	62 (21.9)	0.599
Diuretic, n (%)	612 (30.8)	93 (32.9)	0.493
Digitalis, n (%)	20 (1.0)	1 (0.4)	0.504
Vasodilator, n (%)	104 (5.2)	16 (5.7)	0.776
Insulin, n (%)	247 (12.4)	36 (12.7)	0.923
Oral hypoglycemic, n (%)	799 (40.3)	120 (42.4)	0.518
Hypertension, n (%)	1,699 (85.6)	241 (85.2)	0.857
Diabetes, n (%)	1,067 (53.8)	126 (44.5)	0.004
Dyslipidemia, n (%)	1,298 (65.4)	187 (66.1)	0.841
Current/former smoking, n (%)	759 (38.2)	109 (38.5)	0.948
Prior MI, n (%)	666 (33.6)	100 (35.3)	0.546
Prior PCI, n (%)	275 (13.9)	34 (12.0)	0.459
Prior CABG, n (%)	265 (13.4)	49 (17.3)	0.080
Positive family history of CAD, n (%)	65 (3.3)	7 (2.5)	0.588
Obesity, n (%)	451 (22.7)	55 (19.4)	0.223
Physical inactivity, n (%)	1,040 (52.4)	155 (54.8)	0.484
Prior stroke, n (%)	68 (3.4)	13 (4.6)	0.306
Shock/IABP use, n (%)	8 (0.4)	2 (0.7)	0.360
ACS, n (%)	754 (38.0)	125 (44.3)	0.043
eGFR, mL/min/1.73 m^2^(median [P25; P75])	72.6 [55.7; 91.8]	78.0 [59.7; 100.0]	0.003

ACEi: angiotensin-converting enzyme inhibitor; ACS: acute coronary syndrome; ARB: angiotensin receptor blocker; CABG: coronary artery bypass grafting; CAD: coronary artery disease; CCB: calcium-channel blocker; CIN: contrast-induced nephropathy; eGFR: estimated glomerular filtration rate; IABP: intra-aortic balloon pump; MI: myocardial infarction; PCI: percutaneous coronary intervention; SD: standard deviation.

We fit a logistic regression model including an interaction between contrast type and volume to test whether the effect of contrast type depended on administered volume. The model was adjusted for age, sex, baseline creatinine, congestive heart failure, and ACS ( Table 4 ). The interaction term was not significant (p > 0.05), indicating no effect modification by volume.

**Table 4:** – Logistic models with interaction term for contrast-induced nephropathy

Variables	Interaction model OR (95% CI)	p value	Adjusted interaction model1, OR (95% CI)	p value
Contrast				
Ioxaglate	—	—	—	—
Iodixanol	0.96 (0.75-1.24)	0.773	0.94 (0.73-1.20)	0.618
Contrast volume				
< 150 mL	—	—	—	—
≥ 150 mL	1.07 (0.64-1.71)	0.796	1.07 (0.64-1.71)	0.796
Interaction (Iodixanol × ≥ 150 mL)	1.33 (0.69-2.60)	0.397	1.35 (0.70-2.65)	0.377

^1^Adjusted for sex, age, baseline creatinine, congestive heart failure, and acute coronary syndrome. OR: odds ratio.

After removing the interaction term, neither contrast type nor volume showed an independent association with CIN. As shown in Table 5 , there was a non-significant trend toward higher risk with volumes ≥ 150 mL in the unadjusted model (odds ratio [OR] = 1.24; 95% CI 0.88-1.71; p = 0.202) and in the adjusted model (OR = 1.25; 95% CI 0.89-1.73; p = 0.191). Additional analyses treating volume as a continuous variable likewise demonstrated no significant association with CIN.

**Table 5:** – Additive models for contrast-induced nephropathy

Variables	Additive model, OR (95% CI)	Valor p	Adjusted additive model1, OR (95% CI)	Valor p
Contrast				
Ioxaglate	—	—	—	—
Iodixanol	1.00 (0.80-1.26)	0.974	0.98 (0.78-1.23)	0.857
Contrast volume				
< 150 mL	—	—	—	—
≥ 150 mL	1.24 (0.88-1.71)	0.202	1.25 (0.89-1.73)	0.191

^1^Adjusted for sex, age, baseline creatinine, congestive heart failure, and acute coronary syndrome. OR: odds ratio.

Figures 1 and 2 depict the lack of relationship between contrast volume and change in serum creatinine. Figure 3 shows the volume-by-type interaction effect on CIN, which was non-significant in both the unadjusted (OR = 1.33; 95% CI 0.69-2.60; p = 0.397) and adjusted models (OR = 1.35; 95% CI 0.70-2.65; p = 0.377).

**Figure 1:** – Density of change in serum creatinine by contrast-volume category.

**Figure 2:** – Change in serum creatinine by contrast-volume category. SD: standard deviation.

**Figure 3:** – Incidence of CIN by contrast type and volume category. CIN: contrast-induced nephropathy; OR: odds ratio.

## Discussion

CIN remains a relevant concern in diagnostic and therapeutic procedures that use iodinated contrast, particularly PCI.^9^In this study, contrast type (iso-osmolar vs low-osmolar) was not associated with CIN; in high-risk populations the critical elements appear to be minimizing administered volume and using pre-procedure hydration.

Prior research suggests important roles for both contrast type and volume in CIN risk.^9 , 10^However, we observed no significant differences between iso-osmolar and low-osmolar agents, regardless of volume, noting that only a minority received truly high volumes in this cohort. Earlier studies posited a potential advantage of iso-osmolar agents due to less hemodynamic and renal stress,^11 , 12^but more recent evidence indicates that, in carefully selected patients managed with standardized hydration protocols, agent selection may not meaningfully alter the incidence of CIN. Moreover, osmolarity is not the sole determinant of renal response to contrast.

The absence of between-group differences may reflect the effectiveness of preventive strategies. Intravenous hydration — broadly recommended in guidelines — combined with lower contrast volumes likely attenuated any effect of contrast type on renal function.

Clinical heterogeneity also warrants consideration. Factors such as diabetes, advanced age, and pre-existing kidney disease — although adjusted for — can modulate individual risk of CIN and may contribute to the lack of a global effect.

In practice, contrast agent selection should be individualized, considering clinical conditions, risk profile, and local resource availability. Prospective studies with larger samples and robust designs are needed to confirm these findings and explore potential differences within specific subgroups.

### Limitations of study

Beyond osmolarity and volume, other physicochemical properties of contrast media may influence the development of CIN; therefore, these results should not be extrapolated to agents other than the two evaluated here. In addition, this was a single-center trial, which limits generalizability — particularly to settings that use higher contrast volumes or serve different patient populations.

## Conclusion

Contrast type was not significantly associated with CIN, even among patients exposed to higher contrast volumes.
